# Endocannabinoids in Bladder Sensory Mechanisms in Health and Diseases

**DOI:** 10.3389/fphar.2021.708989

**Published:** 2021-07-05

**Authors:** Stewart Christie, Simon Brookes, Vladimir Zagorodnyuk

**Affiliations:** Discipline of Human Physiology, College of Medicine and Public Health, Flinders Health and Medical Research Institute, Flinders University, Adelaide, SA, Australia

**Keywords:** endocannabinoids, bladder afferents, painful bladder syndrome, overactive bladder, bladder dysfunction

## Abstract

The recent surge in research on cannabinoids may have been fueled by changes in legislation in several jurisdictions, and by approval for the use of cannabinoids for treatment of some chronic diseases. Endocannabinoids act largely, but not exclusively on cannabinoid receptors 1 and 2 (CBR1 and CBR2) which are expressed in the bladder mainly by the urothelium and the axons and endings of motor and sensory neurons. A growing body of evidence suggests that endocannabinoid system constitutively downregulates sensory bladder function during urine storage and micturition, under normal physiological conditions. Similarly, exogenous cannabinoid agonists have potent modulatory effects, as do inhibitors of endocannabinoid inactivation. Results suggest a high potential of cannabinoids to therapeutically ameliorate lower urinary tract symptoms in overactive bladder and painful bladder syndromes. At least part of this may be mediated via effects on sensory nerves, although actions on efferent nerves complicate interpretation. The sensory innervation of bladder is complex with at least eight classes identified. There is a large gap in our knowledge of the effects of endocannabinoids and synthetic agonists on different classes of bladder sensory neurons. Future studies are needed to reveal the action of selective cannabinoid receptor 2 agonists and/or peripherally restricted synthetic cannabinoid receptor 1 agonists on bladder sensory neurons in animal models of bladder diseases. There is significant potential for these novel therapeutics which are devoid of central nervous system psychotropic actions, and which may avoid many of the side effects of current treatments for overactive bladder and painful bladder syndromes.

## Introduction

The endocannabinoid system (ECS) consists of several endocannabinoids and their G-protein coupled receptors (GPCRs) together with synthetizing and degradation enzymes which are present in nearly every bodily tissue including bladder and dorsal root ganglion (DRG) neurons (Merriam et al., 2011; Strittmatter et al., 2012; Bakali et al., 2013; Izzo et al., 2015; Hedlund and Gratzke, 2016; Bjorling and Wang, 2018). The two most-studied endocannabinoids are N-arachidonoylethanolamine (anandamide; AEA) and 2-arachidonoylglycerol (2-AG) which act on cannabinoid receptors (CBRs), CBR1 and CBR2. CBR1 is mainly expressed in nervous tissue whilst CBR2 is expressed on immune tissue and peripheral afferent nerves (Rice et al., 2002; Hillsley et al., 2007; Ruggieri, 2011; Cristino et al., 2020). Both AEA and 2-AG act extensively in the central and peripheral nervous system, affecting pain, mood, feeding behavior, motivation and inflammation (Cristino et al., 2014; Vuckovic et al., 2018). However, in addition to activation of CBR1/CBR2, endocannabinoids and their synthetic analogues can modulate various ion channels including some transient receptor potential (TRP) channels, ligand-gated ion channels and GPCRs (Pertwee, 2010; Christie et al., 2020). Cannabinoids may also act on so-called atypical or cannabinoid-like receptors such as GPR55 and peroxisome proliferator-activated receptors (PPARs) (Bouaboula et al., 2005; Wrobel et al., 2020).

Cannabinoids have been recently demonstrated to suppress pain behavior in various rodent models of inflammatory pain, such as arthritis and inflammatory bowel disease (Romero-Sandoval et al., 2015; Vuckovic et al., 2018). In the bladder endocannabinoids likely regulate the activity of sensory neurons involved in urine storage and micturition (Hedlund, 2014). Further, the ECS may have potential to ameliorate lower urinary tract symptoms such as urgency and pain associated with common bladder disorders, including overactive bladder (OAB) and painful bladder syndromes (PBS) (see recent reviews Hedlund and Gratzke, 2016; Bjorling and Wang, 2018). This mini review focuses on recent data revealing the ability of endocannabinoids to modulate sensory neuron function regulating urine storage and micturition, and their potential to treat OAB syndrome and PBS.

## Effects of Cannabinoids on Micturition: Possible Involvement of Sensory Nerves

**FIGURE 1:**
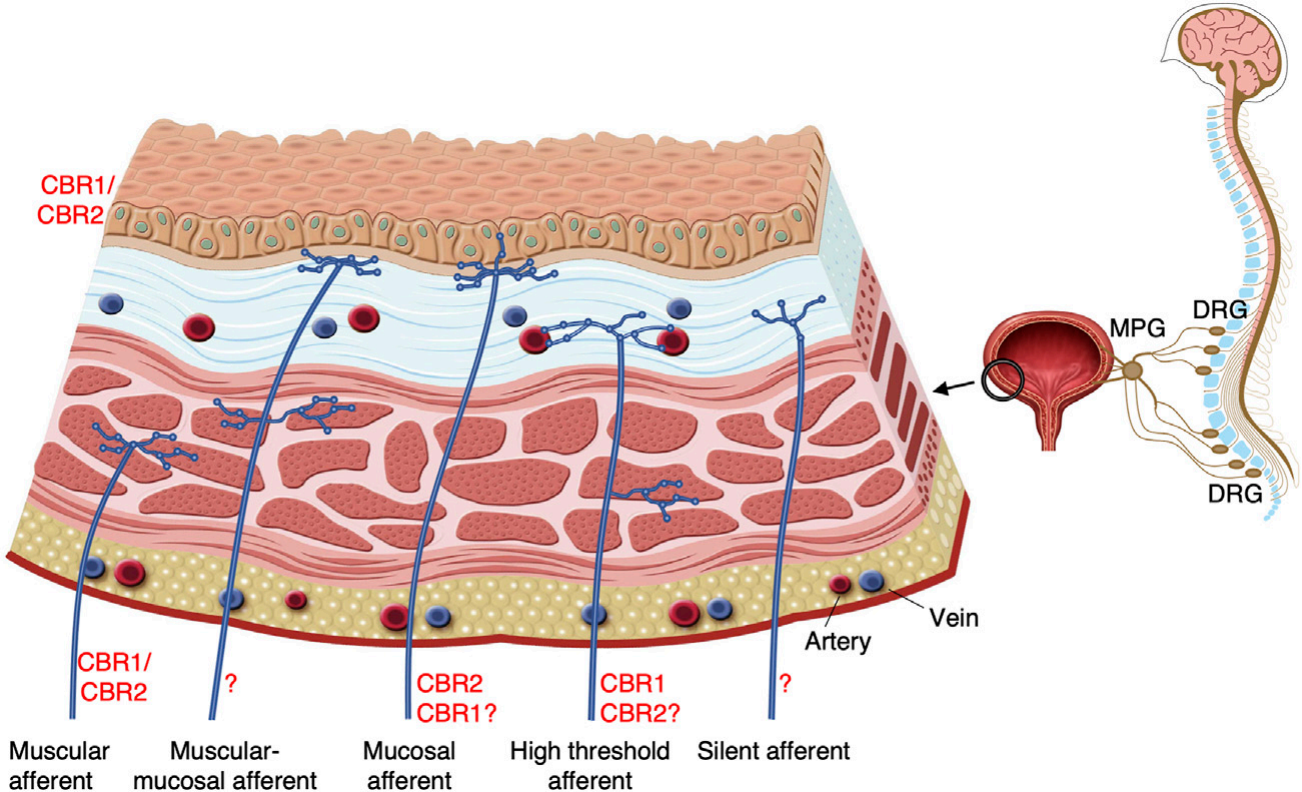
Potential targets of endocannabinoids on bladder sensory nerve endings. At least eight classes of bladder afferents within five major types can be distinguished in the lumbar splanchnic and sacral pelvic neural pathways. These include low-threshold non-encoding and encodings muscular afferents, muscular-mucosal afferents, high- and low-responding mucosal afferents (largely in the lumbosacral DRG innervating evenly all areas of the bladder) and high-threshold muscular and vascular afferents and mechano-insensitive silent afferents (predominantly in the thoracolumbar DRG innervating the base of the bladder) (Xu and Gebhart, 2008; de Groat et al., 2015; Guo et al., 2020). Only five types of bladder afferents illustrated at the figure. These classes/types of afferents have receptive fields with specific structures and locations within the bladder wall and possess specific combinations of ion channels and receptors which regulate their excitability (Zagorodnyuk et al., 2010; Merrill et al., 2016; Grundy et al., 2019a). Both low- and high-threshold afferents may undergo sensitisation as a result of bladder inflammation and thus transmit nociceptive information (Roppolo et al., 2005; Walczak and Cervero, 2011; Yoshimura et al., 2014; Zagorodnyuk et al., 2019). Pelvic low-threshold non-encoding muscular and muscular-mucosal afferents signal mechanical information about micturition and non-painful sensation from the bladder. Low-threshold encoding afferents, high-threshold muscular and vascular afferents, mucosal high-responding and silent afferents probably all detect noxious mechanical and chemical stimuli. These then contribute to pathological states, which result in lower urinary tract symptoms. Potential targets of endocannabinoids in the bladder include CBR1 on the high threshold stretch-sensitive afferents (Walczak and Cervero, 2011); CBR1/CBR2 on the low-threshold distension-sensitive afferents (Aizawa et al., 2014) and CBR2 on the mucosal high-responding afferents (Christie and Zagorodnyuk, 2021). More functional studies are imperative to fully characterise CBR types and their intracellular transduction mechanisms in different classes of bladder afferents.

In the bladder, CBR1 and CBR2 are expressed in urothelial cells, detrusor muscle and nerve fibres (Hayn et al., 2008; Gratzke et al., 2009; Bakali et al., 2013). Despite the expression of CBR1 and CBR2 in the detrusor, endocanabinoids have little (Saitoh et al., 2007) or no effect on detrusor muscle itself (Martin et al., 2000; Gratzke et al., 2009). The role of endocannabinoids in modulating sensory function in the bladder is supported by the expression of both CBR1 and CBR2 on the axons in the sub-urothelium and detrusor muscle (Hayn et al., 2008; Gratzke et al., 2009; Walczak et al., 2009; Bakali et al., 2013; Kim et al., 2017) (Figure 1), where they co-localise with established sensory markers such as calcitonin gene-related peptide (CGRP), TRP vanilloid 1 (TRPV1) and P2X3 purinoreceptors (Gratzke et al., 2009; Walczak et al., 2009; Izzo et al., 2015).

Cystometry studies demonstrate that endocannabinoids can modulate micturition intervals and threshold pressure, both of which may reflect bladder afferent function. However, their role in healthy bladders is inconsistent, most likely due to differences in particular drug used (e.g. endocannabinoids and their synthetic analogues versus drugs modifying endogenous level of endocannabinoids), route of administration and/or species studied (see Table 1). For example, both intravesical and serosal application of AEA dose-dependently decreased micturition interval in anaesthetised rats via TRPV1; an effect blocked by a CBR1 antagonist (Dinis et al., 2004). Conversely, in anaesthetised rats, intravenous infusion of an AEA transport inhibitor (VDM11), which increases tissue concentrations of AEA, increased micturition interval and threshold pressure. This effect was blocked by CBR1 antagonist (AM251) but not CBR2 antagonist (AM630) (Honda et al., 2016). These discrepancies are likely from different administration routes of AEA and VDM11. This may lead to preferential action of AEA on different classes of bladder afferents with different profile of expression of CBR1/CBR2 and/or TRPV1. In conscious rats, intravesical or intravenous administration of the FAAH inhibitor, oleoyl ethyl amide inhibited bladder reflex activity, similar to VDM11. However, these effects were abolished by a CBR2 antagonist (SR144528), whilst a CBR1 antagonist (rimonabant) only reduced effects on threshold pressure (Strittmatter et al., 2012). This agrees with the effect of selective CBR2 agonist, cannabinor which increased micturition intervals and volume when applied intravenously. This indicates involvement of CBR2 in sensory function since cannabinor did not affect nerve-mediated contractions in the isolated rat bladder strips (Gratzke et al., 2009; Gratzke et al., 2010). However, it is unclear which particular class(es) of bladder afferents are responsible for these effects. It is worth mentioning that in rat and human bladders, CBR1 and CBR2 are also expressed in motor cholinergic bladder nerves (Gratzke et al., 2010; Bakali et al., 2013). Further, endocannabinoids pre-synaptically modulate contractility of isolated bladder preparations mainly via CBR1 in species-dependent manner (Martin et al., 2000; Gratzke et al., 2009). Thus, the direct action of endocannabinoids on efferent nerves means that effects on sensory nerves cannot be identified with certainty from cystometry studies. Rather, direct studies of cannabinoid action on bladder afferent firing may be required.

**TABLE 1:** Cannabinoid drugs and their effects on sensory neurons and bladder function.

Drug	Target	Off Target	Species/Model	Dosage	Route	Effect	References
Arachidonyl-2′-chloroethylamide (ACEA)	CBR1		Mouse LPS cystitis *ex vivo*	2.5 mg/kg	Intraperitoneal	No effect on bladder contractions	Tambaro et al. (2014)
			Mouse *in vivo*	100 µM	Intravesical	↓Bladder activity induced by NGF	Wang et al. (2015a)
Anandamide	CBR1/CBR2	TRPV1	Rat CYP cystitis *in vivo*	1–100 µM	Intravesical Serosal	↓Micturition interval via TRPV1 and CBR1 in cystitis	Dinis et al. (2004)
			Rat *in vivo*	100 µM	Intravesical	↓Micturition interval	Gratzke et al. (2009)
			Guinea pig *ex vivo*	30 µM	Organ bath	↑Mucosal afferent mechanosensitivity via TRPV1	Christie, Zagorodnyuk, (2021)
Ajulemic acid	CBR1/CBR2		Rat *ex vivo*	75 nM	Organ bath	↓ATP and capsaicin induced CGRP release via CBR1 and CBR2	Hyan et al. (2008)
AZ12646915	CBR1/CBR2		Mouse *ex vivo*	100 µM	Intravesical	↓Distension-induced firing of high threshold afferents via CBR1	Walczak et al. (2009)
			Mouse CYP cystitis *ex vivo*	100 µM	Intravesical	↓Sensitisation of stretch-sensitive afferents in cystitis via CBR1	Walczak and Cervero, (2011)
Cannabinor	CBR2		Rat *in vivo*	3 mg/kg	Intravenous	↑Micturition interval ↑Micturition volume	Gratzke et al. (2010)

CP55,940	CBR1/CBR2	GPR55	Rat *in vivo*	0.005 mg/kg	Intravesical	↑Micturition interval and threshold pressure	Gratzke et al. (2009)
			Rat in control and acetic acid OAB *in vivo*	0.005 mg/kg	Intravesical	↑Micturition interval and bladder capacity in OAB via CBR1 and CBR2	Bakali et al. (2016a)
GP1a	CBR2		Mice acrolein cystitis *in vivo*	10 mg/kg	Intraperitoneally	↓Decreased mechanical sensitivity in cystitis via CBR2	Wang et al. (2014)
JWH-015	CBR2		Mice LPS cystitis *ex vivo*	5 mg/kg	Intraperitoneal	↓ Bladder inflammation and contractions in cystitis via CBR2	Tambaro et al. (2014)
JWH-133	CBR2		Mouse CYP cystitis *in vivo*	1 mg/kg	Intraperitoneal	↓Bladder inflammation ↓Mechanical sensitivity via CBR2	Liu et al. (2020)

O-1602	GPR55	GPR18	Rat retinyl acetate OAB *in vivo*	0.25 mg/kg	Intraarterial	↓Detrusor activity in OAB	Wrobel et al. (2020)
Oleoyl ethyl amide	FAAH		Rat *in vivo*	0.3 mg/kg	Intravenous	↓Bladder reflex activity via CBR2	Strittmatter et al. (2012)
URB937	FAAH		Rat *in vivo*	1 mg/kg	Intravenous	↓Distension-induced firing and PGE_2_ sensitisation of C-fibers via CBR1 and CBR2	Aizawa et al. (2014)
							Aizawa et al. (2016)
VDMI	AEA transporter		Rat *in vivo*	3–10 mg/kg	Intravenous	↑Micturition interval ↑Bladder capacity via CBR1	Honda et al. (2016)

Abbreviations: CYP – cyclophosphamide, NGF – nerve growth factor, LPS – lipopolysaccharide, FAAH – fatty acid amide hydrolase, AEA – anandamide, CBR – cannabinoid receptors.

## Sensory Innervation of the Bladder

Spinal sensory neurons innervating the bladder play a key role mediating both storage and micturition. They are responsible for bladder sensations, ranging from physiological sensation of filling and fullness through to lower urinary tract symptoms such as urgency and pain (de Groat and Yoshimura, 2009; Merrill et al., 2016). The bladder has a dual sensory innervation, with afferents projecting via sacral pelvic nerves (cell bodies from L5 to S2 DRG) and to lesser extent via lumbar splanchnic nerves (cell bodies from T12 to L2) (Keast and De Groat, 1992; Nandigama et al., 2010). Thinly myelinated Aδ fibre afferents in the pelvic-sacral nerves are essential for the sensation of filling and initiation of the micturition reflex (Fowler et al., 2008). In cats, C-fibre bladder afferents are normally not mechano-sensitive but can be activated by noxious stimuli or acquire mechano-sensitivity during inflammation (Janig and Koltzenburg, 1990). However, in rats and mice, both pelvic Aδ fibre and C-fibre afferents mostly express CGRP and TRPV1 and the majority are mechano-sensitive (Shea et al., 2000; de Groat et al., 2015; Kadekawa et al., 2017).

Sensation from the bladder involves at least eight classes of bladder afferents within five major **types** of afferents (Figure 1). Low-threshold **muscular afferents** are “in series tension receptors” which respond to bladder distension and contraction and have receptive fields in the detrusor (Shea et al., 2000; Zagorodnyuk et al., 2007). They comprise two classes: 1) narrow range afferents, whose firing plateaus at high distension (“non-encoding muscular afferents”) and 2) wide dynamic range afferents (“encoding muscular afferents”), which continue to increase firing across the full range of distension including noxious one (Xu and Gebhart, 2008; Mills et al., 2020). In guinea pigs, muscular afferents are not CGRP-immunoreactive and are unresponsive to capsaicin, a TRPV1 agonist (Zagorodnyuk et al., 2010). However, in mice, most low-threshold stretch-sensitive afferents are capsaicin-sensitive (Daly et al., 2007). Another type of low-threshold afferent, **muscular–mucosal** (or muscular–urothelial) **afferents** are sensitive to both low amplitude stretches and to light mucosal stroking (Zagorodnyuk et al., 2007; Xu and Gebhart, 2008). In contrast to muscular afferents, these afferents are also sensitive to the chemical composition of urine. They are likely to have receptive fields in both the detrusor and in the lamina propria (Zagorodnyuk et al., 2009).

CGRP- and capsaicin-immunoreactive fibres form a dense plexus beneath the urothelium (Fowler et al., 2008; Zagorodnyuk et al., 2010). Most of these belong to stretch-insensitive **mucosal afferents** which can be activated by light mucosal stroking and by chemical stimuli (Zagorodnyuk et al., 2007; Xu and Gebhart, 2008). Their receptive fields consist of simple endings in the lamina propria, close to urothelial cells (Zagorodnyuk et al., 2010; Spencer et al., 2018). Mucosal afferents respond to numerous factors released from urothelial cells, including adenosine triphosphate, nitrogen oxide, and prostaglandins. These mediators are released in response to chemical, thermal and mechanical stimuli (Birder and Andersson, 2013). Two classes of mucosal mechanoreceptors have been distinguished in the guinea pig bladder; 1) capsaicin-sensitive mucosal high-responding afferents, which are activated by a variety of noxious stimuli and 2) mucosal low-responding afferents which respond weakly to mucosal stroking, but may be activated by hypertonic stimuli (Zagorodnyuk et al., 2007).

In many *in vivo* and *ex vivo* studies, high threshold (>15 mmHg or >20 g load) stretch-sensitive mechanoreceptors have been distinguished in the bladder (Sengupta and Gebhart, 1994; Zagorodnyuk et al., 2007; Xu and Gebhart, 2008; Grundy et al., 2019b). They are also activated by blunt probes or stiff von Frey hairs, but not by light mucosal stroking (Xu and Gebhart, 2008; Song et al., 2009). **High-threshold afferents** probably represent a heterogenous group of mechanoreceptors. Some have endings in the detrusor (high threshold muscular afferents) (Zagorodnyuk et al., 2010) while high-threshold “vascular afferents”—have receptive fields associated with blood vessels in the suburothelium (Song et al., 2009), similar to intramural “vascular” mechanoreceptors (previously called “serosal” afferents), found in the intestine (Brookes et al., 2013). Most of the high-threshold afferents in the bladder are capsaicin-sensitive (Nicholas et al., 2017; Zagorodnyuk et al., 2019). In addition, a significant group of bladder afferents (up to 30%) do not to respond to any level of distension and therefore have been called “ **silentafferents** ” or “mechano-insensitive afferents” (Janig and Koltzenburg, 1990; McMahon et al., 1995; Guo et al., 2020). During acute inflammation some of these become spontaneously active and mechanosensitive and may contribute to acute and chronic pain states in cystitis (McMahon et al., 1995; de Groat et al., 2015).

## Direct Effect of Cannabinoids on the Bladder Afferents

To date, relatively few studies have investigated the direct effect of cannabinoids on bladder afferents (Table 1). The non-selective CBR1/CBR2 agonist AZ12646915 reduces distension-evoked firing of high-threshold pelvic afferents in mouse bladder via CBR1 (Walczak et al., 2009). A non-selective cannabinoid agonist, ajulemic acid, reduces ATP- and capsaicin-evoked release of CGRP in the rat bladder; an effect reversed by both CB1 and CB2 antagonists, AM252 and AM630 (Hayn et al., 2008). Intravenous administration of a selective, peripherally restricted fatty acid amide hydrolase (FAAH) inhibitor, URB937, increases the tissue concentration of endocannabinoids and simultaneously decreases distension-induced activity of Aδ and C-fibers in *in vivo* studies in rats. This effect is abolished by CBR1 and CBR2 antagonism, rimonabant and SR144528 (Aizawa et al., 2014). URB937 also reduces the sensitisation of C-fibers induced by intravesical administration of PGE_2_ (Aizawa et al., 2016). It is important to note that endogenous cannabinoids can constitutively downregulate bladder sensory neuron function in rats and mice since both CBR1 (rimonabant and AM251) and CBR2 antagonists (SR144528) alone significantly increase distension-induced firing (Walczak and Cervero, 2011; Aizawa et al., 2014). Distension-induced afferents studied in whole bladder *ex vivo* (Walczak et al., 2009; Walczak and Cervero, 2011) or *in vivo* (Aizawa et al., 2014) likely include both low- and high-threshold muscular and muscular-mucosal classes of bladder afferents. However, in the guinea pigs, the stable analogue of anandamide, methanandamide (up to 30 µM) did not affect stretch-induced firing of muscular-mucosal afferents (Christie and Zagorodnyuk, 2021). The highly selective CBR2 agonist, 4-quinolone-3-carboxamide, inhibits the mechanosensitivity of high-responding mucosal afferents (Christie and Zagorodnyuk, 2021). CBR2 antagonist, SR144528 on its own did not significantly change stroking-induced firing of mucosal afferents. Methanandamide potentiated the mechanosensitivity of mucosal afferents to stroking via TRPV1. In the presence of TRPV1 antagonist (capsazepine), its effect switched to inhibitory (Christie and Zagorodnyuk, 2021). It is still unclear whether excitatory and inhibitory effects of methanandamide involved the same or different CBRs. Overall, these studies indicate potent and species-dependent modulatory effects of endogenous and exogenous cannabinoids on bladder afferents which occurs via CBR1 and CBR2 (see Figure 1). Undoubtedly, more studies are needed to fully elucidate complex direct effects of endocannabinoids and their synthetic agonists on different classes of bladder afferents.

## Cannabinoids and Overactive Bladder

In humans with idiopathic detrusor overactivity (DO), the density of CBR1 on nerve fibres in the suburothelium and detrusor is increased (Mukerji et al., 2010). However, in detrusor sections from DO patients, assessing CBRs in entire detrusor section rather than focusing on nerve fibres, both CBR1 and CBR2 mRNAs and general immunohistochemical staining were increased in the mucosa, but decreased in the detrusor (Bakali et al., 2016b). Experimental obstruction-induced DO in rats is associated with increases in CBR1 expression in the urothelium, detrusor and sacral spinal cord, with no changes in CBR2 (Fullhase et al., 2016; Kim et al., 2017). This highlights difficulties in translating the data obtained in animal models to humans. The effects of endocannabinoids in animal models of OAB are summarised in Table 1. Given the presence of both CBR1 and CBR2 on nerve fibres within the bladder wall and relatively modest effects of CBR activation on bladder motor function, these effects on DO may be mediated by cannabinoid agonists acting on afferent fibres via CBR1 and CBR2. However, secondary effects via urothelial and interstitial cells cannot be ruled out. Recently, evidence has suggested that GPR55, a possible third CBR, may also be a potential target for treatment of overactive bladder since the GPR55 agonist O-1602 significantly reduces DO in rats (Wrobel et al., 2020).

## Cannabinoids and Cystitis

In patients with PBS, the expression of CBR1 in suburothelial, but not detrusor nerve fibres is significantly increased (Mukerji et al., 2010). CBR2 mRNA, but not CBR1 mRNA, increased in acrolein-induced cystitis in rats (Merriam et al., 2008) and lipopolysaccharide (LPS)-induced cystitis in mice (Tambaro et al., 2014). However, in acrolein-induced cystitis in mice, there was no changes in CBR2 expression (Wang et al., 2014). No changes in the expression of CBR2 were reported in the mouse bladder in cyclophosphamide-induced cystitis (Liu et al., 2020). These discrepancies may be due to species differences and/or the method of induction of cystitis.

AEA levels in rat, but not mouse bladder are increased during bladder inflammation (Dinis et al., 2004; Merriam et al., 2011; Wang et al., 2015b; Bjorling and Wang, 2018). Importantly, the non-selective CBR1/CBR2 agonist AZ12646915 reverses the sensitisation of stretch-sensitive afferents in cyclophosphamide-induced cystitis (Walczak and Cervero, 2011). CBR1 on high threshold stretch-sensitive afferents is likely the target of this agonist (Figure 1). The selective CBR1 agonist, arachidonyl-2’-chloroethylamide (ACEA), inhibits the increased bladder activity induced by nerve growth factor (NGF) (Wang et al., 2015a). Further, increasing AEA levels by inhibiting FAAH (Merriam et al., 2011) or using a FAAH knockout (Wang et al., 2015b), decreased pain behaviour and bladder hypersensitivity in rats and mice.

It is important to note that endocannabinoids such as AEA do not only act via CBR1 and CBR2 but can also activate TRPV1 channels, which are pro-nociceptive in the bladder and other tissues (Charrua et al., 2007; Marrone et al., 2017). Indeed, AEA, via activation of TRPV1, contributes to hyperreflexia and hyperalgesia during cyclophosphamide-induced cystitis (Dinis et al., 2004). Therefore, the strategy to increase the level of endogenous cannabinoids appears less attractive since the action of endocannabinoids is complicated by off-target effects on ligand and voltage-gated channels. Further, pharmacological inhibition of FAAH in humans demonstrated low efficacy in reducing chronic pain and had adverse side effects (Feldwisch-Drentrup, 2017; Vuckovic et al., 2018).

Animal models suggest that CBR2 may be a potential target to ameliorate pain and bladder inflammation in cystitis. Selective CBR2 agonists (JWH015, JWH133 and GP1a) reduce the inflammation, pain behavior, and urinary frequency associated with cystitis (Tambaro et al., 2014; Wang et al., 2014; Liu et al., 2020). Whether this is due to reduced inflammation or direct effects on bladder afferents, or both is not clear. A growing body of evidence indicates that CBR2 activation decreases infiltration of inflammatory cells and markers such as leukocytes, interleukins, and tissues necrosis factors in cystitis (Rice et al., 2002; Tambaro et al., 2014; Wang et al., 2014; Liu et al., 2020). This suggests that a reduction in inflammation itself by CBR2 activation may be one of the main causes for a reduction in inflammatory hyperalgesia.

Chemically related to AEA, palmitoylethanolamide (PEA) is a ligand for PPARα (Lo Verme et al., 2005). PEA has significant analgesic effects (Keppel Hesselink and Kopsky, 2015) and, similar to AEA, PEA levels are elevated in cystitis in rats (Merriam et al., 2011; Pessina et al., 2015). PEA may potentiate effects of endocannabinnoids on the CBRs through an “entourage” effect (Pessina et al., 2015). Exogenous PEA reduces viscero-visceral hyperreflexia induced by NGF, via an effect on CBR2 (Farquhar-Smith et al., 2002). However, exogenous PEA may also attenuate bladder pain and inflammation by enhancing the effect of anandamide on CBR1 (Pessina et al., 2015).

Thus, these data demonstrated that both CBR1 and CBR2 agonists are effective in decreasing pain behaviour and bladder hypersensitivity in animal models of PBS. The exact mechanisms of these effects remain to be established. It is still unclear which specific class(es) of bladder afferents contribute the most to analgesic effect of endocannabinoids as well as exact role of immune, urothelial, and other cells within the bladder wall.

## Conclusion

Despite some discrepancies in the expression of CBR1/CBR2 and the effects of exogenous and endogenous cannabinoids on sensory neurons and micturition in naive bladders and in animal models of OAB and PBS, preclinical research has identified the significant potential of cannabinoids as novel treatments for common bladder disorders. CBR2 agonists and/or peripherally restricted synthetic CBR1 agonists may significantly ameliorate lower urinary tract signs/symptoms in animal models of OAB and PBS. They are likely to lack the CNS psychotropic actions and sides effects of current treatments. Use of FAAH and other inhibitors to elevate the level of endogenous endocannabinoids currently appears less attractive since the action of endocannabinoids is complicated by off-target effects on ligand and voltage-gated channels, low efficacy, and possible side effects. This review has highlighted the paucity of basic research studies evaluating the direct effects of cannabinoids on the bladder sensory neurons. At least eight classes of bladder afferents, with very different roles in sensory signaling have been identified to date. To design better strategies for the treatment of lower urinary tract symptoms, further basic research studies are needed to elucidate the mechanisms of direct effects of endocannabinoids and their synthetic agonists on different classes of bladder afferents. This will help establish the precise mechanisms of their analgesic and anti-inflammatory actions in models of bladder diseases.

## Abbreviations

DRG, dorsal root ganglia; MPG, major pelvic ganglion; CBR, cannabinoid receptor.
